# Guava leaf extract as a catalyst for enhanced rabbit health and performance in sub-tropical regions: an updated review

**DOI:** 10.1007/s11250-025-04485-6

**Published:** 2025-05-28

**Authors:** A. H. Abd El-Aziz

**Affiliations:** Department of Animal Wealth Development, Faculty of Veterinary Medicine, Damanhour University, Damanhur, Egypt

**Keywords:** Guava leaf, Growth performance, Health, economic, Immune, Pathogenic microorganisms

## Abstract

For sustainable intensification of rabbit industry, it is imperative to explore the potential of non-conventional feed resources. The researchers were driven to discover solutions for the shortage of feed in livestock production because of the ongoing rise in costs associated with conventional feed resources. Their efforts were directed at enhancing conventional sources and investigating alternative feed sources, including agricultural and agro-based industrial by-products. Therefore, the review examines the possible advantages of using guava leaf extract (*Psidium guajava*) as an example of non-conventional feed additive to improve the growth performance and overall health of commercially raised rabbits. Guava leaf extract contains a high concentration of bioactive substances, such as antioxidants, vitamins, and vital minerals. These substances have demonstrated advantageous effects on the feed efficiency, growth rate, and overall health of rabbits. This review article provides a thorough analysis of the current understanding and updates the knowledge about the physiological effects of adding guava leaf extract to rabbit diets, with a specific emphasis on its economic benefits. The findings demonstrated notable enhancements in feed conversion efficiency, growth rate, and immune system functionality, coupled with reductions in the total lipids levels and pathogenic microorganisms. Potential avenues for future investigation encompass the refinement of dosage, examination of synergistic impacts with other dietary constituents, and implementation of extended studies to validate the enduring advantages.

## Introduction

Over the past decade, there was a witnessed increase in the rabbit industry due to the large meat yield of the animals with their high reproductive efficiency, rapid growth rate, compact sizes were previously reported (Abd El-Aziz et al. 2020, 2022, 2024a, b). Rabbit production also offers several advantages in the context of environmental sustainability, particularly due to the species'efficient feed conversion capacity. The sustainability of meat production systems is largely influenced by their biological efficiency, which is closely associated with the animal's ability to convert dietary inputs into edible meat (Cesari et al. 2018). Notably, rabbits can convert approximately 20% of the protein they consume into muscle tissue comparable to that of poultry, which ranges from 22 to 23% (Jiang et al. 2020; Kumar et al. 2025). This protein conversion efficiency in rabbits surpasses that of many conventional livestock species, such as beef cattle (8–12%) and pigs (16–18%). Such high efficiency in protein utilization not only supports economic productivity but also contributes to lowering the environmental footprint of meat production systems. Furthermore, the remarkable adaptability of rabbits to diverse environmental conditions and their ability to thrive on agricultural by-products and food waste further enhance their sustainability profile (Hernández 2008; Sanah et al. 2022). Therefore, promoting rabbit farming could significantly contribute to global food security and offer economic opportunities, particularly in low- and middle-income countries (Kumar et al. 2025). Importantly, this elevated efficiency highlights the need for continuous optimization of rabbit diets through strategic use of feed additives aimed at enhancing growth performance and economic returns. Nevertheless, the growing of the rabbit and poultry sectors in Egypt along with many other developing countries, is hindered by increasing costs and scarcity of traditional feed ingredients (Abd El-Ghany 2024; Abdelghani et al. 2024; El-Manylawi and El-Banna 2013). Vast amounts of byproducts result from the rapid expansion of agro-industrial processing worldwide, resulting in environmental pollution caused by improper disposal methods (Freitas et al. 2021). Yet, these byproducts contain bioactive compounds that have potential to decrease oxidative stress and improve overall health (Quintero-Herrera et al. 2023; Reguengo et al. 2022). It is essential to mitigate environmental risk and economic sustainability by efficiently repurposing these byproducts. The huge volumes of agro-industrial waste in Egypt represent an underutilized resource that can be added to animal diets to mitigate nutritional deficiencies and decrease feed costs (Gaur et al. 2020; Quintero-Herrera et al. 2023). Guava (*Psidium guajava* Linn), widely cultivated plant in the tropical and the subtropical regions; has become an important agro-industrial by product source. Guava leaves are traditionally discarded as waste; however, they are rich, containing bioactive compounds, tannins, flavonoids, saponins, terpenoids, and polyphenols have antioxidant, anti-inflammatory and antimicrobial properties (Kumar et al. 2021; Wang et al. 2021). They also have health benefits. For example, fighting metabolic disease, improving respiratory health and treating infections (Carolino et al. 2022). In addition, guava leaf extract (GLE) has been proven effective in food preservation whereby it prevents bacterial growth or a possible bacterial strain form *E. coli*, *Shigella*, *Staphylococcus* and *Clostridium*, which also extends the shelf life (Raj et al. 2023). In recent years, Guava leaf extract (GLE) has been studied as a natural option to synthetic additives in animal feed, and these studies have brought promising results. For instance, (Kumar et al. 2021) reported that GLE acts as an antioxidant, antimicrobial, anti-inflammatory, anti-diarrheal agent. These properties put GLE in a good position to replace antibiotics in the feed in livestock systems, which is currently a global issue regarding the counteraction with antibiotic resistance in the animal’s production systems (Golovinskaia and Wang 2023; Kumar et al. 2024). Moreover, it has been noticed an increase in growth performance, improved health status, and reduced lipids in broilers supplemented with guava leaf meal (Daing et al. 2021). Similarly, guava byproducts have successfully been added to the diets of finishing lambs up to 30%, exhibiting economic and nutritional benefits without any adverse health problems (Nobre et al. 2020). Despite these promising findings, there are few studies that present the overall impact of GLE in rabbits that provides a range of changes from molecular to physiological levels. While, Morsy et al. (2019) showed that dietary GLE supplementation might significantly enhance growth performance and selected blood biochemical characteristics, nonetheless, key questions relating to action mechanisms, immunity, hormones, and histological alterations resulting from GLE supplementation are still unanswered. Further, no detailed report on the negative effects/risks of using GLE supplement and the potential risks involved have not been presented adequately. By synthesizing and increasing available information on GLE’s effectiveness in rabbit farming, this review intends to fill these gaps. Specifically, it scrutinizes its impact on growth rate, nutrient utilization, blood characteristics, immune system, carcass characteristics and profitability. The review also describes the processes by which GLE possesses antiradical, antioxidant, anti-inflammatory, and immunomodulatory properties and analyzes the consequences to offer a critical perspective of the benefits and hazards of its use.

To gather the relevant literature, we performed a thorough search of peer-reviewed journals, databases, and academic repositories to collect pertinent literature using preset keywords such as Guava, *Psidium guajava*, rabbit diet, guava leaf extract, growth performance, immunity, carcass traits, economic efficiency and health factors. Rigorous inclusion and exclusion criteria were implemented to guarantee the inclusion of only high-quality, relevant studies, so generating a solid basis for our results.

This systematic review aims to elucidate the impact of Guava leaf extract supplements on rabbit nutrition and provide novel insights and recommendations for formulating focused strategies to improve performance, health, and welfare for sustainable rabbit production.

## Nutritional and phytochemical profile of guava

Guava possesses an exceptional array of bioactive compounds. The desiccated pomace is composed predominantly of seeds (94%) and skins (6%) (Abd El-Ghany 2024; Denny et al. 2013; Nicanor et al. 2001). Nutritional research reveals a high crude fiber content (61%), ether extracts (12%), and energy value (1336 kcal/kg), in addition to linoleic acid and other advantageous chemicals (Lira et al. 2011). Moreover, guava's essential oils comprise several substances such as α-pinene, 1,8-cineole, and terpineol, which enhance its antibacterial and antioxidant properties (Ramadan et al. 2009). The ethanolic and methanolic extracts of this plant contain flavonoids (quercetin, guaijaverin), polyphenols, kaempferol and phenolic acids (corosolic and ursolic). They showed that bioactive molecules present in it is capable of exhibiting antibacterial, antioxidant as well as anti-inflammatory properties (Chen and Yen 2007; Nguyen et al. 2023). Discoveries from Psidials L and M and Psipinene contribute to the medicinal array of Guava and show prospects for the further utilization of this fruit (Sherif et al. 2023).

## Mechanism of guava leaf extract

### Antioxidant properties

One of the major guava leaves properties is that they contain phenolic compounds including quercetin, ferulic and gallic in high levels, all of which are effective antioxidants. These compounds scavenge reactive oxygen species (ROS) and decrease oxidative stress which determines growth and immune status of rabbits. Research proves that polyphenols found in the guava leaf extract offer protection to cells against damage, optimize the metabolic rate and increase the immunity of body (Abd El-Ghany 2024; Abdelghani et al. 2024; He and Venant 2004; Kimura et al. 1985).

### Antimicrobial activity

The guava leaves extract is rich in flavonoids and tannins, which have good antimicrobial properties, and which work against many forms of bacteria. Such compounds are effective against pathogenic bacteria agents, including *Escherichia*, *Salmonella* spp., and *Staphylococcus aureus*. The addition of guava leaf extract enhances gut microbiota and the occurrences of enteric infections in rabbits apart from enhancing nutrient digestibility and feed conversion ratio in rabbit diets (Abd El-Ghany 2024; Abdelghani et al. 2024; Chah et al. 2006; Nair and Chanda 2007).

### Anti-inflammatory and hepatoprotective effects

Asiatic acid – a triterpenoids compound of the guava leaves– can decrease inflammation and help to protect the liver. These compounds reduce inflammation and inhibit lipid accumulation in the liver to improve general well-being and economic gains in rabbits (Abd El-Ghany 2024; Abdelghani et al. 2024; Gao Jing et al. 2006; Roy et al. 2006).

### Gut health and nutritional efficiency

The phytochemicals also enable guava leaf extract to create a healthy effect to the gut by eliminating pathogenic bacteria and encouraging generative microbes in its place. This favorable gut condition strengthens feed to meat conversion ratio, meat production rates in rabbits (Abd El-Ghany 2024; Abdelghani et al. 2024; Adeyemi et al. 2009).

### Immune modulation

The immunomodulatory role associated with the consumption of guava leaf extract decreases prevalence of disease and improves health status of rabbits used in intensive production (Abd El-Ghany 2024; Abdelghani et al. 2024).

Taken together, all these mechanisms explain the versatility of the use of guava leaf extract and create a sound scientific foundation for use of the guava leaf extract as a natural feed supplement in rabbit production.

## Physiological effects guava leaf extract as an additive in rabbit diet

**Fig. 1:**
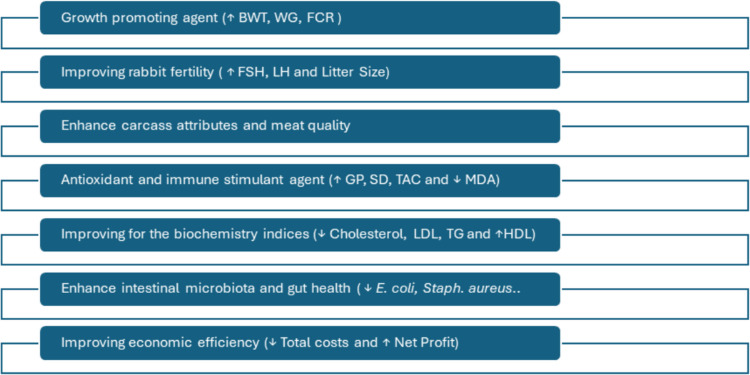
The potential usefulness of using guava leaf extract as a natural non-conventional feed additive in rabbits

Using bioactive compounds from agro-industrial waste promotes growth and animal health; it also diminishes the impact on the environment in the livestock business. Egypt is one main producer of guava globally, and, thus, the need to embrace sustainable and environmentally-friendly practices in the production of guava byproducts (Abdelghani et al. 2024; Zahid et al. 2019). Previous studies have documented that supplementing rabbit and poultry diets with guava leaf extract (GLE) can have various physiological effects as depicted in Fig. 1. Most of these implications are also summarized in Table 1. and illustrated in the following parameters.

**Table 1:** A Comprehensive Summary of Key Studies on Guava Leaf Extract in Rabbits and Poultry

Aspect	Species	Dose/Percentage	Key Effects	References
Growth Performance	Rabbits	30% GU with Kemzyme®	6.6% weight gain improvement compared to the control group; enhanced FCR but no impact on feed intake at 10%−20%	(Ali 2008)
	Rabbits	1–3 ml GLE/kg diet	Significant improvement in final weight, ADG, and FCR at 2–3 ml/kg diet; negligible mortality rates	(Morsy et al. 2019)
	Broilers	1% dried leaves	Improved body weight, weight gain, and FCR with no effect on feed intake	(Mahmoud et al. 2013)
	Rabbits	20% GW diet	Improved body weight gain and performance index compared to control	(Kamel et al. 2016)
	Rabbits	15–20 mg/kg diet	Highest final body weight (FBW), daily weight gain (DWG) improved Feed intake (FI) increased, but FCR was unaffected	(Abdelghani et al. 2024)
Reproductive Performance	Rabbits	20 GLE mg/kg body weight	Enhanced litter size, prolificity rate, FSH, LH levels, and postnatal survival rates	(Dorice et al. 2023)
Carcass Traits	Rabbits	2–3 ml GLE/kg diet	Increased carcass and liver proportions, reduced belly fat, and improved meat dry matter and crude protein content	(Morsy et al. 2019)
	Broilers	2.5%−4.5% guava meal	Reduction in fat content and improved carcass quality	(Rahman et al. 2013)
	Rabbits	16% GBP	No significant differences in carcass dressing or growth; enhanced nutrient digestibility with enzyme supplementation	(Mekkawy et al. 2000)
	Rabbits	GLE15 (15 mg/kg), GLE20 (20 mg/kg) diet	GLE20 group had the highest hot carcass, liver, spleen, and heart weights GLE15 showed intermediate improvements No significant changes in lung, fur, kidney, cecum, or gastrointestinal tract weights	(Abdelghani et al. 2024)
Blood Parameters	Rabbits	2–3 ml GLE/kg diet	Reduced LDL, triglycerides, and cholesterol; increased HDL and antioxidant capacity (TAC)	(Morsy et al. 2019)
	Broilers	1% dried leaves	Reduction in lipid metabolites; improved HDL concentrations	(Mahmoud et al. 2013)
	Rabbits	Ethanolic GLE at dose 200 mg/ml in distilled water	Enhanced HDL, reduced LDL and triglycerides	(Olaniyan 2017)
	Rabbits	GLE15 (15 mg/kg), GLE20 (20 mg/kg) diet	GLE20 increased packed cell volume (PCV) and mean corpuscular volume (MCV) Reduced triglycerides, cholesterol (total, HDL, LDL), and improved VLDL Lower AST and ALT levels in GLE-treated groups	(Abdelghani et al. 2024)
Immunity and Antioxidants	Rabbits	GLE15 (15 mg/kg), GLE20 (20 mg/kg) diet	GLE20 improved IgG (63.84%) and IgA (26.41%) over control Lymphocytes increased, neutrophils and eosinophils decreased in GLE groups Elevated SOD and TAC levels in GLE groups, with the greatest increase in GLE20 Lower ROS and MDA levels in GLE-treated rabbits	(Abdelghani et al. 2024)
Economic Efficiency	Rabbits	5% DGW or DOC	Improved profitability with lower feed costs	(Waheed et al. 2019)
	Rabbits	20% GW diet	Reduced production expenses; improved net profit and economic efficiency	(Kamel et al. 2016)
Antimicrobial & Gut Health	Broilers	0.04%−0.06% guava extract	Comparable efficacy to vaccination in *Eimeria tenella* prevention; enhanced gut health	(Rattanaphol and Rattanaphol 2009)
	Rabbits	20% GW diet	Improved gut health with reduced harmful bacteria and enhanced nutrient absorption	(Alvarez et al. 2007)
	Broilers	1.4% tannin composition	Suppression of *E. coli* and improved gastrointestinal health	(Mailoa et al. 2014)
	Rabbits	GLE15 (15 mg/kg), GLE20 (20 mg/kg) diet	GLE15 increased villous height and branching with elongated villous epithelium GLE20 further enhanced these features, showing more pronounced improvements in intestinal tissue structure	(Abdelghani et al. 2024)

GU: Guava by-product; GW: Guava waste; GLE: Guava leaf extract; GBP: Guava by-product meal; FCR: Feed conversion ratio; ADG: Average daily gain; TAC: Total antioxidant capacity; DGW: Dried guava waste; DOC: Dried olive cake

### Growth, reproductive performance, and economic efficiency

The promising effects of guava leaf extract (GLE) and its by-products on growth performance, reproductive indices and economic efficiency of animals have been widely investigated, especially in rabbits and broilers. Bioactive compounds like flavonoids, saponins, tannins, and terpenoids, found in Guava, enhance metabolic activity, nutrient absorption, and gastrointestinal health (Kumar et al. 2021; Pandey and Shweta 2011).

#### Growth performance

Abdelghani et al. (2024) reported a dose-dependent enhancement of growth performance in GLE-supplemented rabbits. Rabbits administered 20 mg/kg of GLE (GLE20 group) had a 12.51% higher FBW (final body weight) than control animals (GLE0), whereas lower (15 mg/kg of GLE; GLE15) dosage increased final body weight as well (FBW in GLE15; 9.07% higher FBW than GLE0). These enhancements were suggested to be mediated by better nutrient bioavailability through metabolic economy and intestine capacity improvement brought about by GLE's bioactive compounds. These results may be justified through the significantly higher daily weight gain (DWG) in GLE-supplemented groups, whereas feed conversion ratio (FCR) was stable, indicating further efficient nutrient use without elevated feed intake levels. Likewise, Ali (2008), reported that the feeding of 30% guava by-product meal (GU) alone or in combination with the Kemzyme® (30%) replacing clover hay increased body weight gain (6.6%), when compared to control rabbits. Despite lower feed intake, the FCR improved in the supplemented groups. The supplementation of the guava by-products may have optimized growth performance with a reduction in feed cost. Subsequently, Morsy et al. (2019) studied the influence of different doses of ethanolic guava leaf extract (1, 2 and 3 ml/kg of feed) on APRI line rabbits. G rabbits received 3 ml kg − 1 of GLE, resulting in the greatest FBW and average daily gain (ADG) and significantly improved FCR. This showed that high dose GLE supplementation improves performance indices in comparison with their baseline values, without adversely affecting feed intake. Mahmoud et al. (2013) also showed that when 1% dried guava leaves incorporated in rabbit diet, weight gain and FCR improved in broilers, and the same benefits have been observed with guava by-products. Ogega et al. (2022), added guava fruit processing by-products to broiler diets at 2.5%, 5%, and 7.5%. However, the study reported that broilers fed levels of guava waste greater than 7.5% in the diet have levels of weight gain and feed intake that were below those of the control diet. Overall, these results indicated the effective use of guava waste as a partial replacement of conventional feed ingredients without compromising the overall broiler’s performance, contributing to a more sustainable method of livestock production.

#### Reproductive performance

Guava leaf extract also appears to favorably influence reproductive characteristics. Dorice et al. (2023), who conducted the experiment using a dose of 0, 10, 20, and 30 mg/kg of aqueous guava leaf extract administered to female rabbits, reported that the aqueous guava leaf extract at a dosage of 20 mg significantly increased litter size, fertility rates, FSH, and LH concentrations. Higher doses of GLE also improved postnatal survival, highlighting GLEs potential to improve reproductive outcomes and offspring viability.

#### Economic efficiency

Few studies have answered the questions about the economic consequences of use of guava leaf extract in rabbit’s diet. Morsy et al. (2019) assessed the effects of different levels of ethanolic guava leaf extract on growth parameters, haematological profiles and cost of feed/kg gain of 80 APRI line rabbits. 3.0 ml/kg dose caused highly significant changes in proportions of carcass and its economic value in all groups, and it was shown that the rabbits receiving it had decidedly the best yield out of the 148.6% to 100% of controlled group. Furthermore, Kamel et al. (2016) worked with the use of rabbit diets containing guava waste (GW) with or without the addition of organic acids or Mannan oligosaccharide. They had lower costs of production and higher economic returns from their respective supplemented organic acid groups when rabbits received 20% GW. These results correlated with previous literature (Ani and Ugwuowo 2011; El-Deek et al. 2009a; Lira et al. 2009), affirming the cost-efficient approach of the feeding inputs, while productivity was not significantly impacted. Regarding the substitution of alfalfa by 5% of dried guava waste (DGW) or olive cake (DOC), Waheed et al. (2019) assessed the economic viability of substituting alfalfa in rabbit diets with 5% dried guava waste (DGW) or olive cake (DOC) using real economic data. DGW or DOC improved BWG and profit, whereas feeding both DGW and DOC in a single diet was less effective. The results were comparable to previous study Kamel et al. (2016) demonstrated that feeding chickens 20% guava waste meal (GW) supplemented with organic acids or Mannan oligosaccharide (MOS) produced higher (p < 0.05) body weight gain (BWG), feed conversion ratio (FCR) and performance index (PI) than control diets. These findings underscore guava’s potential as a cost-effective feed additive for improving farm profitability.

#### Bioactive compounds and antioxidant properties

Guava contains high concentrations of bioactive components, including saponins, flavonoids, tannins, and vitamin C, that have been associated with growth-promoting animal bioactivities (Bikrisima et al. 2014; Pandey and Shweta 2011). They also aid in nutrient absorption and gut health and have antioxidant benefits. Flavonoids such as quercetin and tannins are a key player in suppressing pathogenic bacteria and curtailing oxidative stress which in turn promotes animal health and productivity (Abdelghani et al. 2024; Kumar et al. 2021; Mailoa et al. 2014).

#### Sustainability and environmental benefits

Using guava processing waste as an alternative feed ingredient can help solve key challenges that pervade livestock production such as feed scarcity and waste disposal. Utilization of guava by-products in animal feeding not only lowers production costs but also contributes to environmental sustainability through waste reduction and enhancement of food security in resource-limited regions (Ogega et al. 2022).

Collectively, both Guava leaf extracts and their by-products are promising agents for improving growth performance, reproductive traits, and for economic efficiency in livestock production. The inclusion of GLE in the diets of rabbits and broilers results in higher nutrient utilization, lesser feed cost and sustainable practices without affecting the health and production of the animal. Further studies on guava by-products on supplementation levels and other benefits under different livestock systems should be conducted.

### Carcass traits and meat quality

As mentioned by Abdelghani et al. (2024), revealed that dietary supplementation affected carcass traits in rabbits in general and more particularly at the dose of GLE20. Rabbits in this group had more hot carcass weight and relative organ weight including liver, spleen and heart than those in GLE0. Hence, this recent study demonstrated that the GLE can advance organ development and enhance SSL carcass yield. A highly significant improvement was observed in GLE15 (15 mg/kg) indicating that the effect was dose dependent and therefore higher doses would produce comparable results. Nonetheless, other carcass characteristics such as lung weight, fur weight, kidney weight, GIT weight, and cecum length had not shown any changes due to GLE supplementation. In a similar manner. Morsy et al. (2019) similarly reported significant improvements in carcass proportions and liver weights in rabbits supplemented with guava leaf extract at doses of 2 and 3 ml/kg diet. These groups also showed notable increases in abdominal fat, stomach, small intestine, and cecum proportions, with reductions in GIT weights. Additionally, dietary inclusion of GLE at higher doses enhanced dry matter (DM), crude protein (CP), and ether extract (EE) content in rabbit meat, suggesting its role in improving meat quality. The negative correlation between ether extract and abdominal fat proportions suggests GLE’s potential for reducing fat deposition, aligning with findings by Rahman et al. (2013), who reported decreased fat content in broilers supplemented with guava leaf meal. In broilers, guava supplementation demonstrated consistent improvements in meat quality and fat reduction. (Medina et al. 2006) reported that incorporation of 0.04–0.06% GLE caused changes on the meat composition. Similarly, El-Deek et al. (2009a) have also stated that abdominal fat was significantly lower in the 8% raw or processed guava by-products fed broilers compared to other dietary levels or basal diets. broilers fed up to 12% guava waste (GUW) grew as well as control birds fed corn-soybean meal diets, and had similar feed intake, weight gain, feed conversion ratio and carcass yields (Lira et al. 2009). These results suggested that guava by-products could be used as natural sources of feed supplements to increase yield and quality of broiler meat production without any adverse effects. Furthermore, Mekkawy et al. (2000) showed that substituting up to 16% of alfalfa hay with GBP, supplemented with enzymes or exposed to gamma irradiation, had no significant impact on growth, feed intake, feed efficiency, or carcass dressing percentages. However, GBP diets improved digestive efficiency, as indicated by enhanced digestibility of organic matter (OM), crude protein (CP), and ether extract (EE). These results suggest that GBP can partially replace traditional feed ingredients without compromising growth performance or carcass traits. Studies incorporating guava by-products with dietary additives, such as Mannan oligosaccharide (MOS) or organic acids, demonstrated favorable impacts on carcass characteristics. Kamel et al. (2016) stated that supplementing rabbits with guava leaf extract and by-products in diets increases carcass characteristics of rabbits and meat quality characteristics. These benefits are dose-dependent and primarily affect vital organs and fat deposition. Furthermore, guava by-products serve as cost-effective feed additives, supporting sustainable animal production systems while maintaining or improving growth performance and carcass yield. Future studies should explore optimized supplementation strategies to maximize these benefits across different species.

### Hematological and biochemical parameters

Guava Leaf Extract (GLE) supplementation has demonstrated significant impacts on hematological and biochemical parameters in rabbits, reflecting its potential as a dietary supplement for improving health and productivity. Abdelghani et al. (2024) found that 20 mg/kg GLE (GLE20) improved packed cell volume (PCV) and mean corpuscular volume (MCV), indicating enhanced oxygen-carrying capacity. Immunological markers also improved, with decreased neutrophil and eosinophil counts, alongside increased lymphocyte levels, suggesting a bolstered immune response. Lipid profile analysis revealed reduced triglycerides, total cholesterol, LDL, and improved HDL and VLDL levels, indicating a cardioprotective effect. Liver health markers (AST and ALT) improved in GLE20, demonstrating hepatic protection, while other parameters, such as glucose and total protein, remained unaffected, underscoring GLE’s safety. Morsy et al. (2019), corroborated these findings in a study with 80 APRI rabbits supplemented with GLE at doses of 1, 2, and 3 ml/kg. Higher doses significantly reduced plasma triglycerides, cholesterol, and LDL, while increasing HDL levels. These changes align with earlier findings (Crespo and Esteve-Garcia 2002; Mahmoud et al. 2013) that guava extract lowers lipid metabolites by impeding hepatic lipogenesis. GLE also enhanced antioxidant defense, as evidenced by increased total antioxidant capacity (TAC) and reduced malondialdehyde (MDA) levels. These effects were attributed to phenolic and volatile compounds in guava, which exhibit potent antioxidant and hypoglycemic activities (Ramadan et al. 2009). Furthermore, After complete blood count (CBC) analysis, GLE supplementation did not significantly alter hemoglobin (Hb), red blood cell (RBC) count, or platelet levels, though these values showed a positive trend and remained within normal ranges (Moore et al. 2015). Notably, white blood cell (WBC) counts and heterophil proportions significantly decreased in groups receiving 2–3 ml/kg GLE, suggesting improved immune modulation. This was accompanied by slight increases in monocytes, basophils, and eosinophils. These findings align with studies showing guava’s immunomodulatory effects in poultry and rabbits (Abdelghani et al. 2024; Kamel et al. 2016). In female rabbits, Dorice et al. (2023) also demonstrated that aqueous GLE supplementation significantly enhanced reproductive parameters. At a dose of 20 mg/kg, there were notable increases in litter size, prolificity rate, FSH, and LH levels, as well as improved postnatal survival rates. Biochemical markers such as protein levels also improved, supporting fertility and pregnancy outcomes.

The biochemical parameters assessed in multiple studies, including liver enzymes (AST, ALT), total protein, albumin, globulin, and lipid profiles, remained within normal ranges across all GLE-treated groups, indicating no adverse effects (Kamel et al. 2016; Mekkawy et al. 2000). The stability of these parameters highlights GLE’s safety as a dietary additive. Taken together, the supplementation of GLE improves hematological, lipid, and antioxidant profiles in rabbits, while maintaining safety and stability in biochemical parameters. These findings suggest that GLE, particularly at higher doses (2–3 ml/kg or 20 mg/kg), is a potent dietary additive for enhancing health, productivity, and reproductive outcomes in rabbits. Further research could optimize its usage and explore its broader applications in livestock nutrition.

### Immunity and anti-oxidant related parameters

Guava by-products have been explored as natural antioxidant feed additives in poultry diet that improves positive production efficiencies and meat (Oliveira et al. 2018). Guava extract may enhance oxidative defenses through the increase of enzymes such as catalase (CAT) and polyphenol oxidase. (Almulaiky et al. 2018). Also, incorporation of dried guava leaves and/or olive oil in to the diets has increased the activity of superoxide dismutase (SOD) and glutathione peroxidase (GPx-1) enzymes in broilers to a significant level (Mahmoud et al. 2013). Similar findings were noted Langerudi et al. (2022), the supplementation of 5 mg/kg guava essential oil in broiler diets led to the highest ever measured GPx-1 enzyme activity. Furthermore, supplementation of aqueous guava extract for four weeks have shown the enhancement of the major endogenous antioxidant’s glutathione reductase in streptozotocin induced diabetic animals (Ramadan et al. 2009). Guava extract has been associated with the enhancement of the body antioxidant system, decrease in lipid peroxidation as well as the reduction of the levels of ROS. Similarly, supplementation of 5 mg/kg guava essential oils enhanced the activity of SOD, GPx-1, and glutathione-S-transferase, because of the ability of the guava essential oils in the supplementation to become potent free radical-scavenging and antioxidant nutrient (Chen and Yen 2007). Chen and Yen (2007) noted that the antioxidant capacity was positively associated with the phenolic content and free radical scavenging activity of the guava leaf extract. Elements of phenolic compounds that induced notable antioxidant actions in guava include gallic acid, pyrocatechol, taxifolin, ellagic acid, and ferulic (Farag et al. 2020; Haida et al. 2011). The other important antioxidant flavonoids present in guava are quercetin, hesperidin, kaempferol, rutin, catechin and apigenin. Also, the bioactive compounds such as kaempfertin, isoquinoline, corilaginoline alkaloids and others have been separated using high-performance liquid chromatography (Taha et al. 2019). Flavonoids found in the guava extracts include guavin and its derivatives, vitexin, orientin, and vincasinin II, IV and V. Other compounds in the extracts include benzene-1,2-diol, 2-O-methyl guanosine, 5-bromo-8-(5-nitrosalicylidene amino) quinoline hydrochloride, guavanoic acid, protocatechu Together with phenols, flavonoids, pentacyclic triterpenoids, they have antioxidant and antidiabetic activity of guava fruits (Kumar et al. 2021). Also, it was noted that quercetin flavonoids oxidation of peroxidase to 3,4-dihydroxybenzoic acid improves the antioxidant and antibacterial properties of guava. The flavonoid and polysaccharide content of guava leaves act as antioxidants by suppressing ROS generation, hindering amylase and α-glucosidase activity, and lowering lipid peroxidation and cell death (Kim et al. 2016; Kumar et al. 2021). In addition, the subsequent preparation of silver nanoparticles with crude polysaccharides derived from guava showed considerable ability to scavenge the DPPH radicals and ABTS radical cations (Wang et al. 2017). More recently, Abdelghani et al. (2024), demonstrated that supplementing rabbit diets with guava leaf extract (GLE) significantly influenced immunity, antioxidant activity, and hormonal responses, with the effects varying based on the dose of supplementation. The highest levels of improvement in immunoglobulins, antioxidant markers, and hormonal regulation were observed in the 20 mg/kg GLE (GLE20) group, and the second highest in the 15 mg/kg GLE (GLE15) group, compared to the control group (GLE0, no GLE was administrated). GLE20 caused the highest increase in IgG and IgA, showing increases of 63.84% and 26.41%, respectively, when compared to control. The antioxidant markers, including superoxide dismutase (SOD) and total antioxidant capacity (TAC) were significantly enhanced in the GLE-treated groups, and the greatest enhancement was observed at GLE20. Further, levels of reactive oxygen species (ROS), as well as malondialdehyde (MDA), were significantly decreased in rabbits treated with 20 mg/kg and 15 mg/kg GLE, suggesting reduced oxidative stress. The hormonal response to glucose maximization showed the GLE20 group having significantly more triiodothyronine (T3) than other groups for metabolism acceleration. Compared with the other groups, the measurement of serum cortisol decreased significantly in both GLE20 and GLE15 groups, while the greatest reduction was in the GLE20 group (P ≤ 0.01), indicating less stress. Our data confirm the dose-dependent action of GLE, and that supplementation level of 20 mg/kg was the best to improve immunity, antioxidant activity and hormonal balance in the growing rabbits.

### Intestinal morphology and gut health

Rabbits supplemented with 15 mg/kg GLE (GLE15) showed significant histological enhancements. In the intestine, villous height increased, with moderate branching of villi and elongated villous epithelium. Moreover, the liver tissue in this group revealed an increased number of binucleated cells while maintaining normal structural integrity of the portal vein and bile ducts. Rabbits receiving 20 mg/kg GLE (GLE20) exhibited even greater improvements. These findings highlight the dose-dependent impact of GLE supplementation on the intestinal and hepatic histomorphology of growing rabbits, with the 20 mg/kg dose demonstrating the most significant enhancements in tissue structure and cellular activity (Abdelghani et al. 2024). It has been examined that guava extracts possess high antibacterial action against the different types of bacteria. Furthermore, Pereira et al. (2023), showed that among the bacterial strains, both the sensitive and the resistant Gram-positive was neutralized by aqueous guava leaf extracts. For instance, extracts prepared with methanol interrupts the growth of Methicillin-resistant *Staphylococcus aureus* and *S. epidermidis* (Chakraborty et al. 2018; Richard et al. 2013), while water soluble extracts reduce *Streptococci* species (Shafiei et al. 2016). The antibacterial effects of guava were attributed to their phenolic compounds, guavins A-D, psydiolic acid, and flavonoids such as quercetin and saponins (Huang et al. 2021; Kumar et al. 2021). Extracts from guava roots (Liharaka Kidaha et al. 2013) and fruits (Iha et al. 2008), further highlight the plant’s antimicrobial potential. Guava leaf extracts, prepared using methanol, ethanol, or water, demonstrate robust antimicrobial effects against *E. coli*, *Pseudomonas aeruginosa*, and *S. aureus* (Mailoa et al. 2014; Patel et al. 2019). Lectins in guava prevent *E. coli* adhesion to intestinal walls, thus reducing infection risks (Okemo et al. 2001). Additionally, guava essential oils exhibit strong activity against *P. aeruginosa*, *Streptococcus faecalis*, *Bacillus subtilis*, and *S. aureus* (Soliman et al. 2016).

## Precautions and dosage considerations

While Guava leaves extract (GLE) is beneficial, excessive supplementation may lead to adverse effects; thus, careful dosage is essential (Morsy et al. 2019). Previous studies documented the toxicological profile of guava extracts, particularly the methanolic bark extract, had shown that acute oral administration at high doses (5000 mg/kg) in Wistar rats caused no mortality or observable signs of toxicity, indicating a high median lethal dose (LD50). However, subacute toxicity studies involving daily administration of doses up to 1000 mg/kg over 28 days revealed significant alterations in body weight, organ weights, and biochemical parameters. Histopathological evaluations indicated mild liver inflammation at the highest dose, suggesting that repeated high doses could result in minor organ toxicity (Manekeng et al. 2019).

## Drawbacks of guava byproducts for poultry feeding

### High fiber content

Crude fiber including lignin and pectin components is massive in Guava byproducts. Fiber is a necessary element for gut health in rabbits, albeit too much fiber — particularly indigestible fiber — can restrict nutrient availability and energy density within the diet. In rabbits and poultry, this may lower feed intake and enhance growth performance (El-Deek et al. 2009b). Soluble fiber pectin may form viscous gels in the gastrointestinal tract, delaying gastric emptying and nutrient absorption, which in turn may reduce growth rate (Forman and Schneeman 1980).

### Variability in nutrient composition

Guava byproducts have a widely varying nutritional profile depending on the variety of the plant, processing techniques and inclusion of different parts of the fruit from pulp to seeds and peels. This variability has important implications for balanced diets formulation, as rabbits and poultry need precise nutrient proportions to avoid deficiencies or toxicities (Kamel et al. 2016; Lira et al. 2009). For example, guava byproducts contain crude protein (CP) at 7.5 to 10.1%, which is not sufficient to meet the requirements of growing rabbits without supplementation (Kamel et al. 2016).

### Negative impact on growth performance

According to (El-Deek et al. 2009b), high levels of guava by-product feed are bulky, leading to decreased feed intake and thus weight gain for poultry. Fiber is an important facilitator of gut motility and maintains motility preventing disorders but excess levels dilute diet energy and may overwhelm the rabbits and poultry digestive capacity particularly in growing rabbits.

### Potential digestive challenges

The guava byproduct contains soluble and insoluble fibers, which have different impacts on poultry digestion. Specific soluble fibers like pectin slow down the rate of digestion by coating the gut lining and interfering with enzyme activity. Insoluble fiber is required for gut motility, but it is also a physical diluent, which can reduce consumption of other nutrients (Choct 2015).

### Reduced nutrient digestibility

Guava by-products have low digestibility of crude fibers and other nutrients. Hindgut fermenters, such as rabbits, depend on the microbial fermentation of fiber to take full advantage of fiber intake in their diet, but this is limited by the breakdown of fiber on the high lignin content in guava byproducts, thus decreasing feed efficiency (Tejeda and Kim 2021).

### Risk of reduced palatability

Although lower inclusion levels of guava by-products have been shown to improve feed palatability, at higher inclusion levels palatability may be negatively impacted resulting in reduced feed intake. This is particularly pronounced in younger poultry with immature digestive systems (Guimarães 2007).

## Conclusion and future recommendations

Recent evidence suggests that guava leaf extract (GLE) has the potential to be a versatile, non-conventional feed additive that can enhance rabbit production systems. This provides a natural solution to enhance growth performance, carcass quality, immune function and economic efficiency and environmental sustainability. Several studies confirm key findings that supplementing rabbit diets with GLE at doses of 15–20 mg/kg diet optimizes health and productivity without compromising safety. The antioxidant and antimicrobial properties of it further improve gut health, nutrient absorption and metabolic efficiency.

Nevertheless, these promising results raise a number of critical areas that need to be explored. Future research should focus on:

### Optimal dosage and synergistic effects

It is important to determine the most effective GLE dosage in terms of various rabbit breeds and production systems. The levels of guava byproduct can be incorporated in diet up to a maximum level of ≤ 5% to avail the fiber benefits but a higher amount can also lead to malabsorption of few calories, which in turn leads to their deficiency. Additionally, it could be investigated to what extent GLE benefits can be amplified through synergies with other natural additives, for example probiotics, organic acids or other prebiotics or synbiotics.

### Long-term safety and toxicological studies

To determine the long-term impact of GLE supplementation on rabbit health and performance and to identify any potential risks at higher dosages, the safety and efficacy of GLE supplementation must be assessed.

### Molecular mechanisms and genomic studies

A better understanding of the molecular and genomic mechanisms by which GLE affects growth, immunity, and metabolic pathways will allow for more precision use of GLE in rabbit nutrition.

### Environmental and economic impact analysis

The benefits of GLE in feed systems incorporate reduced feed costs, waste management and carbon footprint can be quantified in comprehensive life cycle assessments.

### Application in diverse livestock systems

Validation of the cross-species applicability of GLE as a sustainable feed additive can be achieved through expanding research to other livestock species. Collectively, by filling these gaps, GLE can become a key component of sustainable rabbit production systems that contribute to global efforts of food security, environmental mitigation and economic viability in the livestock industry.

## Data Availability

Not applicable.

## References

[CR1] Abd El-Aziz AH et al (2020) Influence of multi-enzyme preparation supplemented with sodium butyrate on growth performance blood profiles and economic benefit of growing rabbits. J Anim Physiol Anim Nutr 104:186–195 10.1111/jpn.13227 31657058

[CR2] Abd El-Aziz AH et al (2022) Fructooligosaccharide supplementation boosts growth performance, antioxidant status, and cecal microbiota differently in two rabbit breeds. Animals 12:1528 35739865 10.3390/ani12121528 PMC9219445

[CR3] Abd El-Aziz A, Elfadadny A, Abo Ghanima M, Cavallini D, Fusaro I, Giammarco M, Buonaiuto G, El-Sabrout K (2024a) Nutritional value of oregano-based products and its effect on rabbit performance and health. Animals 14(20):3021. 10.3390/ani14203021 39457951 10.3390/ani14203021 PMC11505053

[CR4] Abd El-Aziz AH, El-Sabrout K, Ghanima MA (2024b) Sodium butyrate supplementation for improving poultry and rabbit performance. Trop Anim Sci J 47(2):252–264

[CR5] Abd El-Ghany WA (2024) The impact of dietary guava (Psidium guajava L.) on some livestock production systems. CABI Rev 19. 10.1079/cabireviews.2024.0018

[CR6] Abdelghani IG et al (2024) Dietary supplement guava leaf extract regulates growth, feed utilization, immune function, nutrient digestibility and redox regulation in growing rabbits. Trop Anim Health Prod 56:325 39361143 10.1007/s11250-024-04126-4 PMC11450086

[CR7] Adeyemi OS, Akanji MA, Oguntoye SA (2009) Ethanolic leaf extract of Psidium guajava: phytochemical and trypanocidal activity in rats infected with Trypanosoma brucei brucei. J Med Plants Res 3:420–423

[CR8] Ali NGM (2008) Physiological responses of growing california rabbits to guava by-products supplementation. J Anim Poult Prod 33:3241–3253

[CR9] Almulaiky Y et al (2018) Assessment of antioxidant and antibacterial properties in two types of Yemeni guava cultivars. Biocatal Agric Biotechnol 16:90–97

[CR10] Alvarez JL et al (2007) Effects of type and level of fibre on digestive physiology and performance in reproducing and growing rabbits. World Rabbit Sci 15:09–17

[CR11] Ani AO, Ugwuowo LC (2011) Response of weaner rabbits to diets containing graded levels of processed velvet beans (Mucuna pruriens). Afr J Biotech 10:14984–14989

[CR12] Bikrisima SHL, Mahfudz LD, Suthama N (2014) Production capacity of broiler chickens fed red guava fruit meal as source of natural antioxidant. Jurnal Ilmu Dan Teknologi Peternakan 3:69–75

[CR13] Carolino MV, Purnamasari L, Dela Cruz JF (2022) The antibacterial properties of Psidium guajava leaf extract as a wound healing agent of laboratory animals: a review. Biotropika J Trop Biol 10:154–160

[CR14] Cesari V et al (2018) Environmental impact of rabbit meat: the effect of production efficiency. Meat Sci 145:447–454 30055437 10.1016/j.meatsci.2018.07.011

[CR15] Chah KF et al (2006) Antibacterial and wound healing properties of methanolic extracts of some Nigerian medicinal plants. J Ethnopharmacol 104:164–167 16226414 10.1016/j.jep.2005.08.070

[CR16] Chakraborty S et al (2018) Antimicrobial activity of Cannabis sativa Thuja Orientalis and Psidium Guajava Leaf Extracts against Methicillin-Resistant Staphylococcus Aureus. J Integr Med 16:350–357 30120078 10.1016/j.joim.2018.07.005

[CR17] Chen H-Y, Yen G-C (2007) Antioxidant activity and free radical-scavenging capacity of extracts from guava (Psidium guajava L.) leaves. Food Chem 101:686–694

[CR18] Choct M (2015) Fibre-Chemistry and functions in poultry nutrition. In: LII Simposio Científico de Avicultura, Málaga, vol 28, pp 113–119

[CR19] Crespo N, Esteve-Garcia E (2002) Nutrient and fatty acid deposition in broilers fed different dietary fatty acid profiles. Poult Sci 81:1533–1542 12412920 10.1093/ps/81.10.1533

[CR20] Daing MI et al (2021) Growth performance, nutrient utilization, blood indices and immunity of broiler chicks fed diets with graded level of condensed tannins containing Psidium guajava leaf meal. Anim Nutr Feed Technol 21:327–340

[CR21] Denny C et al (2013) Guava pomace: a new source of anti-inflammatory and analgesic bioactives. BMC Complement Altern Med 13:1–7 24063346 10.1186/1472-6882-13-235 PMC3849652

[CR22] Dorice AK et al (2023) Biochemical parameters and reproductive traits in female rabbits (oryctolagus cuniculus) exposed to psidium guajava leaf aqueous extract. J Anim Reprod Biotechnol 38:151–157

[CR23] El-Deek AA et al (2009a) Utilization of guava by-products in broiler finisher diets. Egypt Poult Sci J 29:53–79

[CR24] El-Deek AA et al (2009b) Guava by-product meal processed in various ways and fed in differing amounts as a component in laying hen diets. Int J Poult Sci 8:866–874

[CR25] El-Manylawi MA, El-Banna HM (2013) Effect of feeding date stone meal supplemented with Allzyme® on performance of growing New Zealand Rabbits. Egypt J Anim Prod 50:103–109

[CR26] Farag RS et al (2020) Phytochemical screening and antioxidant activity of some medicinal plants’ crude juices. Biotechnol Rep 28:e00536 10.1016/j.btre.2020.e00536 PMC7559852 33088732

[CR27] Forman LP, Schneeman BO (1980) Effects of dietary pectin and fat on the small intestinal contents and exocrine pancreas of rats. J Nutr 110:1992–1999 6158561 10.1093/jn/110.10.1992

[CR28] Freitas LC et al (2021) From waste to sustainable industry: how can agro-industrial wastes help in the development of new products? Resour Conserv Recycl 169:105466

[CR29] Gao Jing GJ et al (2006) Mechanism underlying mitochondrial protection of asiatic acid against hepatotoxicity in mice. J Pharm Pharmacol 58(2):227–233 16451751 10.1211/jpp.58.2.0010

[CR30] Gaur VK et al (2020) Assessing the impact of industrial waste on environment and mitigation strategies: a comprehensive review. J Hazard Mater 398:123019 32768833 10.1016/j.jhazmat.2020.123019

[CR31] Golovinskaia O, Wang C-K (2023) The hypoglycemic potential of phenolics from functional foods and their mechanisms. Food Sci Human Wellness 12:986–1007

[CR32] Guimarães AADS (2007) Utilização do resíduo de goiaba (Psidium guajava L.) na alimentação de poedeiras comerciais. p 42

[CR33] Haida KS et al (2011) Compostos fenólicos totais e atividade antioxidante de duas variedades de goiaba e arruda phenolic compounds and antioxidant activity of two varieties of guava and rue. Rev Atenção Saúde 9(28):1365

[CR34] He Q, Venant N (2004) Antioxidant power of phytochemicals from Psidium guajava leaf. J Zhejiang Univ Sci A 5:676–683 10.1007/BF02840979 15101101

[CR35] Hernández P (2008) Enhancement of nutritional quality and safety in rabbit meat. In: Proceedings of the 9th world rabbit congress, Verona, Italy, pp 10–13

[CR36] Huang J et al (2021) Chemical constituents of Psidium guajava leaves and their antibacterial activity. Phytochemistry 186:112746 33799191 10.1016/j.phytochem.2021.112746

[CR37] Iha SM et al (2008) Phytochemical study of guava (Psidium guajava L.) with potential antioxidant activity aiming at developing a phytocosmetic formulation. Rev Bras 18:387–393

[CR38] Jiang G et al (2020) Strategies for sustainable substitution of livestock meat. Foods 9:1227 32899106 10.3390/foods9091227 PMC7555167

[CR39] Kamel ER et al (2016) Response of New Zealand rabbits to diet containing guava waste (Psidium guaijava L.): 1. Effect on growth performance, diet digestibility and economic efficiency. Alex J Vet Sci 50(1):24–35

[CR40] Kim S-Y et al (2016) Protective effects of polysaccharides from Psidium guajava leaves against oxidative stresses. Int J Biol Macromol 91:804–811 27296444 10.1016/j.ijbiomac.2016.05.111

[CR41] Kimura S, Tamaki T, Aoki N (1985) Acceleration of fibrinolysis by the N-terminal peptide of alpha 2-plasmin inhibitor. Blood 66:157–160 3159442

[CR42] Kumar GD et al (2024) Effect of concentrate feed containing guava waste meal on growth and haemato-biochemical profile of ram lambs. Indian J Small Rumin 30:69–74

[CR43] Kumar P et al (2025) Improving quality and consumer acceptance of rabbit meat: prospects and challenges. Meat Sci 219:109660 39312855 10.1016/j.meatsci.2024.109660

[CR44] Kumar M et al (2021) Guava (Psidium guajava L.) leaves: nutritional composition, phytochemical profile, and health-promoting bioactivities. Foods 10(4):752. 10.3390/foods10040752 10.3390/foods10040752 PMC8066327 33916183

[CR45] Langerudi MT, Youssefi MR, Tabari MA (2022) Ameliorative effect of Psidium guajava essential oil supplemented feed on chicken experimental coccidiosis. Trop Anim Health Prod 54:120 35229230 10.1007/s11250-022-03117-7

[CR46] LiharakaKidaha M, Alakonya AE, Nyende AB (2013) Bioactivity determination of methanol and water extracts for roots and leaves of Kenyan Psidium guajava L landraces against pathogenic bacteria. Springerplus 2:1–7 25674419 10.1186/2193-1801-2-670 PMC4320225

[CR47] Lira RC et al (2009) Inclusion of guava wastes in feed for broiler chickens. Rev Bras Zootec 38:2401–2407

[CR48] Lira RC et al (2011) Chemical composition and energy value of guava and tomato wastes for broilers chickens at different ages. Rev Bras Zootec 40:1019–1024

[CR49] Mahmoud R, Ibrahim D, Badawi M (2013) Effect of supplementation of broiler diets with guava leaves and/or olive oil on growth, meat composition, blood metabolites and immune response. Benha Vet Med J 25:23–32

[CR50] Mailoa MN et al (2014) Antimicrobial activities of tannins extract from guava leaves (Psidium guajava L.) on pathogens microbial. Int J Sci Technol Res 3:236–241

[CR51] Manekeng HT et al (2019) Evaluation of acute and subacute toxicities of psidium guajava methanolic bark extract: a botanical with in vitro antiproliferative potential. Evid Based Complement Alternat Med 2019:8306986 31885665 10.1155/2019/8306986 PMC6927054

[CR52] Medina E et al (2006) Comparison of the concentrations of phenolic compounds in olive oils and other plant oils: correlation with antimicrobial activity. J Agric Food Chem 54:4954–4961 16819902 10.1021/jf0602267

[CR53] Mekkawy SH, El-Faramawy AA, Zakaria SM (2000) Influence of guava by-product, enzyme supplementation and gamma irradiation on performance and digestive utilization of fattening rabbits. Egypt J Radiat Sci Appl 13:139–149

[CR54] Moore DM, Zimmerman K, Smith SA (2015) Hematological assessment in pet rabbits: blood sample collection and blood cell identification. Clin Lab Med 35:617–627 26297408 10.1016/j.cll.2015.05.010

[CR55] Morsy WA, Younan GE, El-Gabry HE (2019) Effect of dietary guava (Psidium guajava l) leaf extract supplementation on productive performance, blood parameters and carcass traits of growing rabbits. Egypt J Nutr Feeds 22:183–192

[CR56] Nair R, Chanda S (2007) In-vitro antimicrobial activity of Psidium guajava L. leaf extracts against clinically important pathogenic microbial strains. Braz J Microbiol 38:452–458

[CR57] Nguyen PD et al (2023) Isolation of quercetin-3-O-sulfate and quantification of major compounds from Psidium guajava L. from Vietnam. J Food Compos Anal 115:104928

[CR58] Nicanor AB et al (2001) Guava seed protein isolate: functional and nutritional characterization. J Food Biochem 25:77–90

[CR59] Nobre PT et al (2020) The impact of dietary supplementation with guava (Psidium guajava L.) agroindustrial waste on growth performance and meat quality of lambs. Meat Sci 164:108105 32145601 10.1016/j.meatsci.2020.108105

[CR60] Ogega EB et al (2022) Effects of inclusion of guava fruit processing by-product in broiler diets on performance. Livestock Res Rural Dev 34:92. Retrieved May 21, 2025, from http://www.lrrd.org/lrrd34/10/3492ogeg.html

[CR61] Okemo PO et al (2001) The kill kinetics of Azadirachta indica A. Juss. (Meliaceae) extracts on Staphylococcus aureus, Escherichia coli, Pseudomonas aeruginosa and Candida albicans. Afr J Sci Technol 2:113–118

[CR62] Olaniyan MF (2017) Cholesterol lowering effect of guava leaves (Psidium guajava) extract on egg yolk induced hypercholesterolaemic rabbits. J Biol Nat 7:24–27

[CR63] Oliveira MDD et al (2018) Antioxidant effect of the guava byproduct in the diet of broilers in the starter phase. Rev Bras Zootec 47:e20160290

[CR64] Pandey A, Shweta M (2011) Antifungal properties of Psidium guajava leaves and fruits against various pathogens. Pharmaceut Biomed Sci J 13(16):1–6

[CR65] Patel P, Joshi C, Birdi T, Kothari V (2019) Anti-infective efficacy of Psidium guajava L. leaves against certain pathogenic bacteria. F1000Research 8:12 10.12688/f1000research.17500.1 PMC6468707 31031967

[CR66] Pereira GA et al (2023) Antimicrobial activity of Psidium guajava aqueous extract against sensitive and resistant bacterial strains. Microorganisms 11:1784 37512956 10.3390/microorganisms11071784 PMC10383264

[CR67] Quintero-Herrera S et al (2023) Turning food loss and waste into animal feed: a Mexican spatial inventory of potential generation of agro-industrial wastes for livestock feed. Sustain Prod Consum 41:36–48

[CR68] Rahman Z et al (2013) Effect of guava (Psidium guajava) leaf meal on production performances and antimicrobial sensitivity in commercial broiler. J Nat Prod 6:177–187

[CR69] Raj MSA et al (2023) Nutritional composition, mineral profiling, in vitro antioxidant, antibacterial and enzyme inhibitory properties of selected Indian guava cultivars leaf extract. Pharmaceuticals 16(12):1636. 10.3390/ph16121636 38139763 10.3390/ph16121636 PMC10747950

[CR70] Ramadan M, Manal Khaled FEM, Abdel Razak HF (2009) Investigation of the chemical composition, antioxidant activity and hypoglycemic effect of the Egyptian guava leaves volatiles. J Arab Soc Med Res 4:137–148

[CR71] Rattanaphol M, Rattanaphol N (2009) Efficacy of crude extrac from guava leaf (Psidium quajava Linn.) for prevention of coccidiosis in broilers inoculated with Eimeria tenella. Proceedings of the 47th Kasetsart University Annual Conference, Kasetsart. Kasetsart University, 83–90

[CR72] Reguengo LM et al (2022) Agro-industrial by-products: valuable sources of bioactive compounds. Food Res Int 152:110871 35181119 10.1016/j.foodres.2021.110871

[CR73] Richard FT, Joshua AT, Philips AJ (2013) Effect of aqueous extract of leaf and bark of guava (Psidium guajava) on fungi Microsporum gypseum and Trichophyton mentagrophytes, and bacteria Staphylococcus aureus and Staphylococcus epidermidis. Adv MedPlant Res 1:45–48

[CR74] Roy K, Kamath V, Asad M (2006) Hepatoprotective activity of Psidium guajava L. leaf extract. Indian J Exp Biol 44:305–311 16629373

[CR75] Sanah I, Boudjellal A, Becila S (2022) Descriptive analysis of rabbit meat marketing parameters in the north-east of Algeria. World Rabbit Sci 30:163–180

[CR76] Shafiei Z et al (2016) Antibacterial and anti-adherence effects of a plant extract mixture (PEM) and its individual constituent extracts (Psidium sp., Mangifera sp., and Mentha sp.) on single-and dual-species biofilms. PeerJ 4:e2519 27761322 10.7717/peerj.2519 PMC5068394

[CR77] Sherif AE et al (2023) Psidium guajava L.: Chemical composition and protective effects of a leaf extract against ethanol-induced cardiotoxicity. S Afr J Bot 162:334–341

[CR78] Soliman FM et al (2016) Comparative study of the volatile oil content and antimicrobial activity of Psidium guajava L. and Psidium cattleianum Sabine leaves. Bull Fac Pharm Cairo Univ 54:219–225

[CR79] Taha TF et al (2019) In vitro bio-medical studies on Psidium guajava leaves. Plant Arch 19:199–207

[CR80] Tejeda OJ, Kim WK (2021) Role of dietary fiber in poultry nutrition. Animals 11:461 10.3390/ani11020461 PMC7916228 33572459

[CR81] Waheed N et al (2019) The effects of dried guava waste and dried olive cake as substitutes for alfalfa on rabbit farm profit. Mansoura Vet Med J 20:15–20

[CR82] Wang L et al (2017) Characterization of silver nanoparticles biosynthesized using crude polysaccharides of Psidium guajava L. leaf and their bioactivities. Mater Lett 208:126–129

[CR83] Wang D et al (2021) Chemical composition and protective effect of guava (Psidium guajava L.) leaf extract on piglet intestines. J Sci Food Agric 101:2767–2778 33140438 10.1002/jsfa.10904

[CR84] Zahid N et al (2019) Advances in guava cultivation. Achieving sustainable cultivation of tropical fruits. Burleigh Dodds Science Publishing, 411–434

